# Inhibiting mevalonate pathway enzymes increases stromal cell resilience to a cholesterol-dependent cytolysin

**DOI:** 10.1038/s41598-017-17138-y

**Published:** 2017-12-06

**Authors:** Sholeem Griffin, Giulio Preta, Iain Martin Sheldon

**Affiliations:** 1 Swansea University Medical School, Swansea University, Swansea, SA2 8PP United Kingdom; 2 Institute of Biochemistry, Vilnius University, Sauletekio 7, Vilnius LT-10257, Lithuania

## Abstract

Animal health depends on the ability of immune cells to kill invading pathogens, and on the resilience of tissues to tolerate the presence of pathogens. *Trueperella pyogenes* causes tissue pathology in many mammals by secreting a cholesterol-dependent cytolysin, pyolysin (PLO), which targets stromal cells. Cellular cholesterol is derived from squalene, which is synthesized via the mevalonate pathway enzymes, including HMGCR, FDPS and FDFT1. The present study tested the hypothesis that inhibiting enzymes in the mevalonate pathway to reduce cellular cholesterol increases the resilience of stromal cells to PLO. We first verified that depleting cellular cholesterol with methyl-β-cyclodextrin increased the resilience of stromal cells to PLO. We then used siRNA to deplete mevalonate pathway enzyme gene expression, and used pharmaceutical inhibitors, atorvastatin, alendronate or zaragozic acid to inhibit the activity of HMGCR, FDPS and FDFT1, respectively. These approaches successfully reduced cellular cholesterol abundance, but mevalonate pathway enzymes did not affect cellular resilience equally. Inhibiting FDFT1 was most effective, with zaragozic acid reducing the impact of PLO on cell viability. The present study provides evidence that inhibiting FDFT1 increases stromal cell resilience to a cholesterol-dependent cytolysin.

## Introduction

Health and the ability to counter pathogenic microbes depends on an organism’s immunity and resilience^1,2^. Immunity, or resistance, is the ability to reduce the pathogen burden by killing infecting microbes. Resilience, or tolerance, is the ability to limit the impact of pathogens on health, by tolerating a given microbial burden^2–4^. The ability to tolerate pathogenic bacteria largely depends on the resilience of the host’s tissue cells to damaging bacterial virulence factors. Cholesterol-dependent cytolysins are a common virulence factor secreted by pathogenic bacteria, and they have a high affinity for cholesterol in the plasma membrane of mammalian cells, where they form 30–50 nm diameter pores^5,6^. These pores allow leakage of molecules across the plasma membrane, resulting in cell death and tissue damage. Most cellular cholesterol is located in the plasma membrane of animal cells, where it constitutes almost half of the lipid molecules^7^. Cholesterol synthesis depends on the production of squalene by the mevalonate pathway^8^. The mevalonate pathway enzymes are common drug targets, used to limit cellular cholesterol synthesis for the control of hypercholesterolemia^9^. Here we explored whether inhibiting the mevalonate pathway to reduce cellular cholesterol in tissue cells could also increase their resilience to cholesterol-dependent cytolysins.

*Trueperella pyogenes* is a Gram-positive bacterium found on the skin and mucosa of many animals, and it causes pathology in several tissues, including mucosa, liver, and skin^10^. Postpartum uterine disease in cattle is the most economically important disease associated with *T. pyogenes* infection, typically affecting 20 to 40% of animals after parturition^11–13^. Uterine disease costs the USA and EU dairy industry about $2 billion/year in lost production, infertility, and treatment costs^11^. The presence of *T. pyogenes* correlates with the severity of endometrial pathology, the extent of the subsequent infertility, and infusion of *T. pyogenes* recapitulates the disease^14–16^. *Trueperella pyogenes* causes inflammation and damage of the stromal compartment of the endometrium, once the surface epithelium is breached during parturition. The main virulence factor secreted by *T*. pyogenes is a cholesterol-dependent cytolysin, pyolysin (PLO)^10^. Bovine endometrial stromal cells are highly sensitive to cytolysis caused by PLO^16^. Whilst antimicrobial therapy limits the severity of disease, there are no treatments to increase the ability of tissues to tolerate *T. pyogenes*. The gap in knowledge is how to manipulate cellular resilience to help tissues tolerate pathogens.

**Figure 1:**
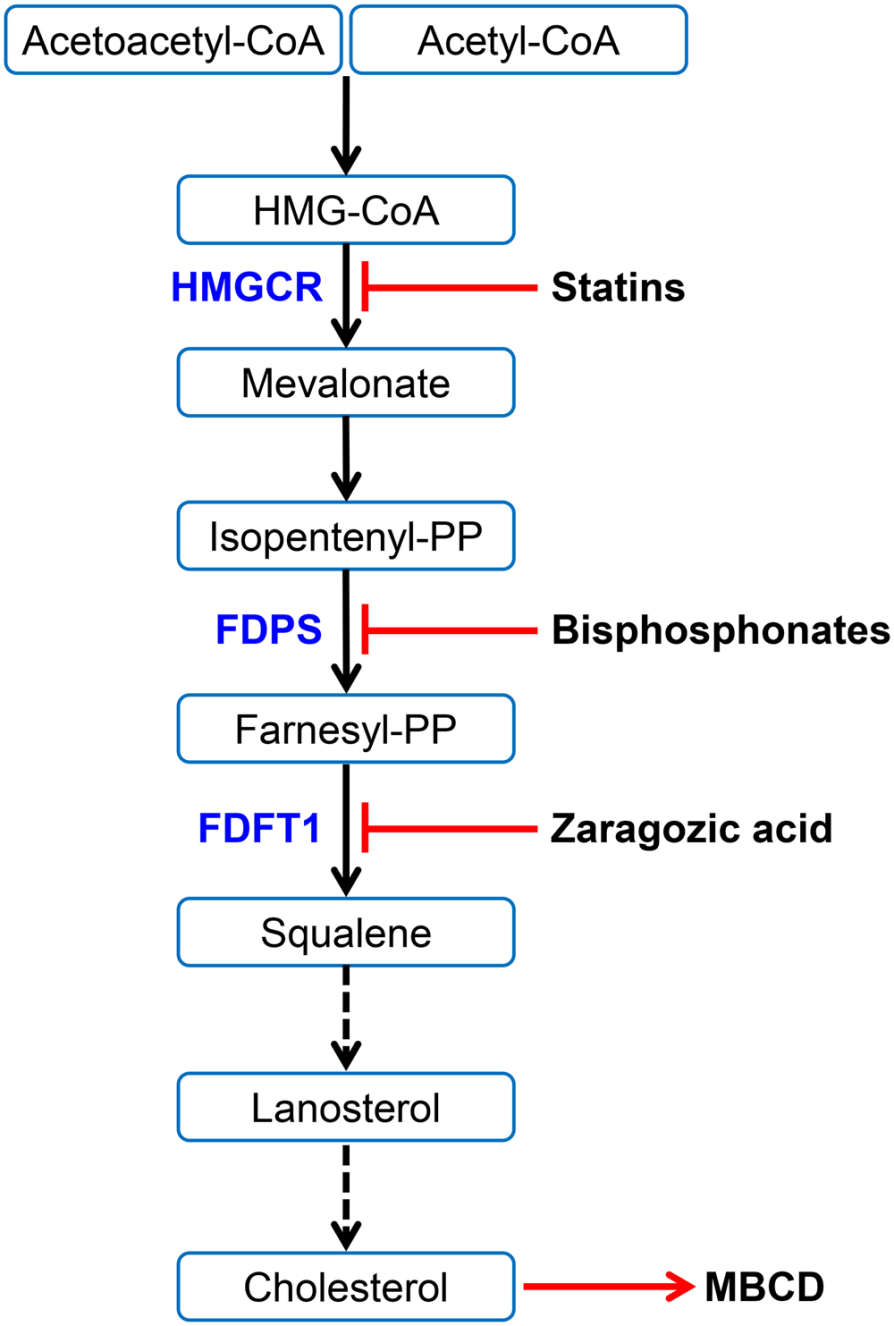
The mevalonate pathway leads to cholesterol synthesis. Acetoacetyl CoA and acetyl CoA are converted to squalene, which is subsequently converted to cholesterol. Important enzymes in the pathway include 3-hydroxy-3-methyl-glutaryl-coenzyme A reductase (HMGCR), farnesyl pyrophosphate synthase (FDPS), and farnesyl diphosphate farnesyltransferase 1 (FDFT1, also commonly called squalene synthase). Each of these enzymes can be inhibited by statins, bisphosphonates, and zaragozic acid, respectively; cholesterol can be depleted using methyl-β-cyclodextrin (MBCD).

There are three pools of cholesterol in the plasma membrane of cells: an essential pool, a sphingomyelin sequestered pool, and a labile pool of cholesterol^17^. The labile cholesterol can be depleted *in vitro* using methyl-β-cyclodextrin (MBCD), which is a cyclic oligosaccharide that binds cholesterol^18,19^. Reducing cellular cholesterol with MBCD increases stromal cell resilience to PLO^16,20^. Cellular cholesterol abundance is highly regulated and depends on the balance amongst cholesterol synthesis, cholesterol efflux, and cholesterol uptake from low-density lipoproteins^9,21^. Cellular cholesterol synthesis uses a series of enzymes, with the mevalonate pathway providing the rate-limiting process^8^. The mevalonate pathway initially condenses two acetyl-CoA molecules to form acetoacetyl-CoA, which are converted to 3-hydroxy-3-methyl-glutaryl-CoA (HMG-CoA) by HMG-CoA synthase, before HMG-CoA reductase (HMGCR, EC 1.1.1.34) yields mevalonate (Fig. 1). A series of enzymes, ending with farnesyl pyrophosphate synthase (FDPS, EC 2.5.1.10), then convert mevalonate to farnesyl pyrophosphate. Farnesyl pyrophosphate is a substrate for several enzymes, but the most important for cholesterol synthesis is farnesyl diphosphate farnesyltransferase 1 (FDFT1, EC 2.5.1.21), which is also called squalene synthase^8,22^.

We reasoned that depleting cellular cholesterol might increase the resilience of cells to PLO. The most common drugs used to reduce hypercholesterolemia regulate the mevalonate pathway include: statins, such as atorvastatin, which inhibit HMGCR; nitrogen-containing bisphosphonates, such as alendronate, which inhibit FDPS; and, zaragozic acid A, derived from fungi, which inhibit FDFT1^9,23–25^. The present study tested the hypothesis that inhibiting enzymes in the mevalonate pathway to reduce cellular cholesterol increases the resilience of stromal cells to PLO.

## Results

### PLO causes cytolysis

To explore the resilience of stromal cells to cholesterol-dependent cytolysins, we chose to use primary bovine endometrial stromal cells (BESC) because they are the main target of *T. pyogenes* ^16^. In addition, to examine whether cellular resilience mechanisms are conserved across species, we used telomerase-immortalized human endometrial stromal cells (HESC)^26^. Using pure populations of stromal cells removes potential confounding effects of immune cells, and helps focus on mechanisms that impact tissue cell resilience, rather than immunity^2,4^. Recombinant pyolysin protein (PLO) was generated and purity was confirmed using SDS-PAGE (Supplementary Fig. S1), as described previously^16,27,28^. The specific activity of PLO was 628,338 HU/mg protein, as determined using a hemolysis assay, and there was very little endotoxin contamination (1.5 EU/mg protein).

**Figure 2:**
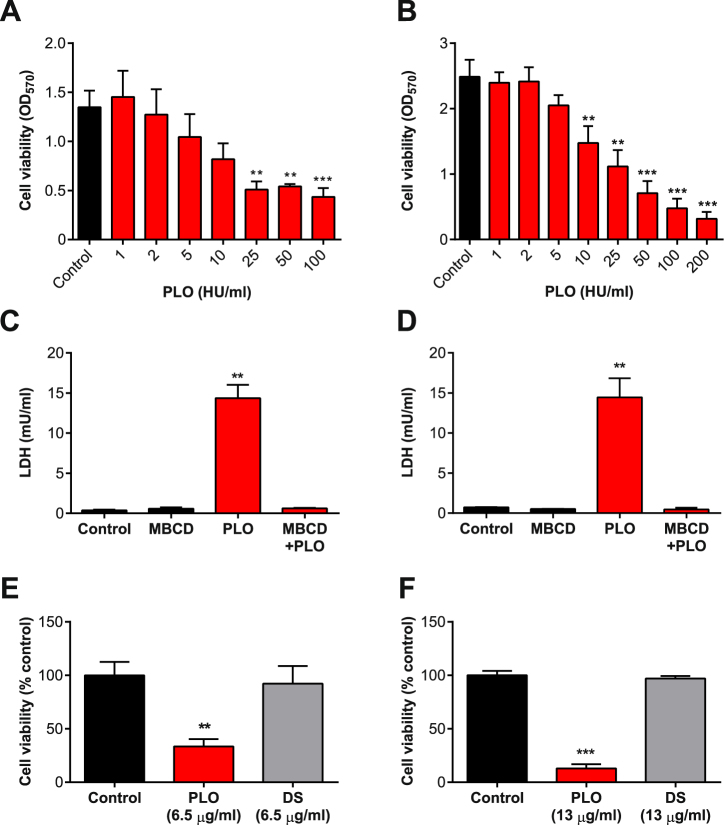
The effect of PLO on stromal cells. (**A**) BESC isolated from 3 animals, and (**B**) HESC from 3 independent passages were challenged for 2 h with control medium (■), or media containing the indicated concentrations of PLO (), and cell viability evaluated by MTT assay. Data are presented as mean (SEM), and analyzed by one-way ANOVA with Dunnett’s multiple comparison post-hoc test; values differ from control, **P < 0.01, ***P < 0.001. (**C**) BESC isolated from 5 animals, and (**D**) HESC from 3 independent passages were grown in control serum-free medium or medium containing methyl-β-cyclodextrin for 24 h to deplete cholesterol, and then challenged for 2 h with control medium (■), or media containing PLO () using 100 HU/ml for BESC and 200 HU/ml for HESC, and leakage of LDH was measured in cell supernatants. Data are presented as mean (SEM), and analyzed by one-way ANOVA with Dunnett’s multiple comparison post-hoc test; values differ from control, **P < 0.01. (**E**) BESC isolated from 3 animals, and (**F**) HESC from 3 independent passages, were challenged for 24 h with control serum-free medium (■), or media containing PLO () or DS-PLO (DS, ), and cell viability evaluated by MTT assay. Data are presented as mean (SEM) percent viability of control, and analyzed by one-way ANOVA with Dunnett’s multiple comparison post-hoc test; values differ from control, **P < 0.01, ***P < 0.001.

The BESC and HESC were cultured in 24-well plates until 70% confluent and then challenged directly with PLO because, unlike most cholesterol-dependent cytolysins, PLO is spontaneously active without the addition of thiol reducing agents^6,10^. To limit the potential effects of cellular uptake of cholesterol or serum binding to PLO, serum-free media were used as described previously^20,29^. After challenging the cells for 2 h with a range of concentrations of PLO, cell viability was assessed by the mitochondria-dependent reduction of 3-(4,5-dimethylthiazol-2-yl)-2,5-diphenyltetrazolium bromide (MTT) to formazan in viable cells^30^. In this colorimetric assay we defined 100% viability as the OD_570_ measurement for cells in control medium, and 0% viability as the OD_570_ for cells lysed using Triton X-100. We assumed that a reduction in OD_570_ was a reflection of cytoxicity, as described previously for experiments using cholesterol-dependent cytolysins^16,20,31^. The correlation between MTT OD_570_ measurements and the number of live cells was confirmed previously using trypan blue exclusion and counting the number of live cells using a hemocytometer^16^. The percentage cell viability was calculated as the OD_570_ of PLO-treated cells relative to OD_570_ values for cells in control medium. The viability of BESC and HESC decreased in a PLO concentration-dependent manner (Fig. 2A,B). For subsequent experiments, we selected a 2 h challenge with 100 HU/ml PLO for BESC and 200/ml HU PLO for HESC.

We confirmed the utility of challenging BESC and HESC with 100 and 200 HU/ml PLO, respectively, by measuring the leakage of lactate dehydrogenase (LDH) from the cytosol into the supernatant, which is a proxy for the formation of pores in plasma membranes by cholesterol-dependent cytolysins^32^. There was increased abundance of LDH in the supernatants of both BESC and HESC challenged with PLO, compared with control (Fig. 2C,D). To verify that PLO activity is cholesterol-dependent, we also depleted cellular cholesterol by treating cells with MBCD, which binds to cholesterol and prevents the formation of pores by cholesterol-dependent cytolysins^16,18,19,33^. When BESC or HESC were treated with MBCD for 24 h, and then challenged with PLO for 2 h, there was no significant increase in supernatant LDH concentrations, compared with control (Fig. 2C,D).

As a control, to verify the cytolytic activity was caused by PLO, we used a mutant PLO protein, which has a protein-stabilizing disulphide bridge (DS-PLO), that binds to plasma membranes but does not form pores^28^. Challenging cells with PLO caused a marked reduction in viability of both cell types, whereas an equivalent amount of DS-PLO did not cause cytolysis (Fig. 2E,F). Taken together, these data provide evidence that BESC and HESC are sensitive to the cholesterol-dependent cytolysin PLO.

### RNA interference of *FDFT1* increases cellular resilience to PLO

**Figure 3:**
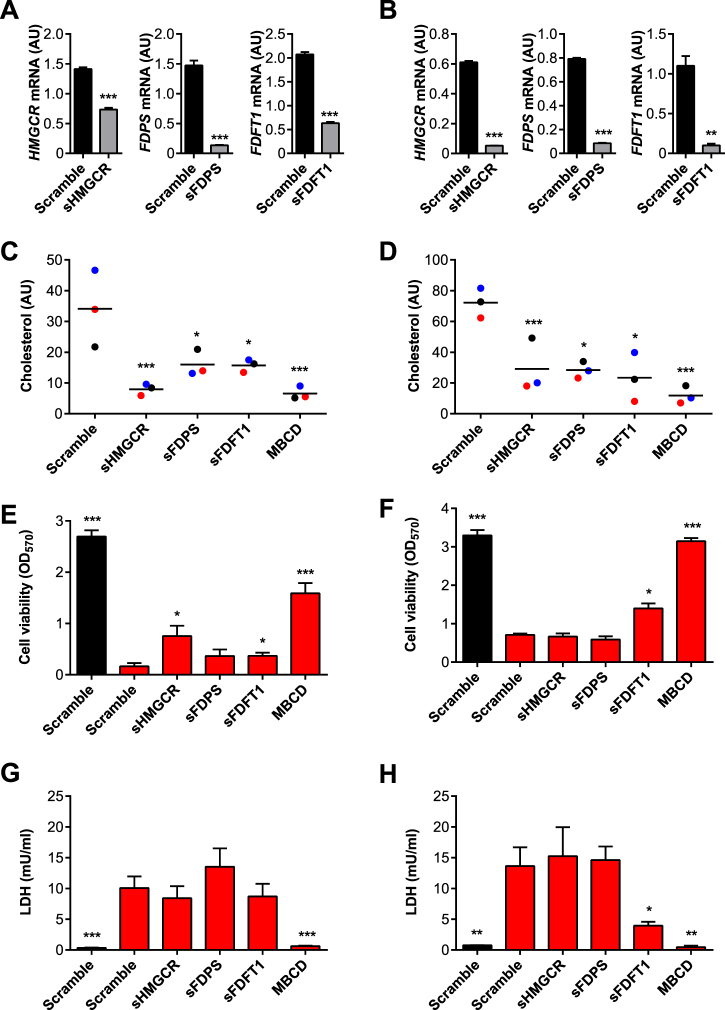
RNA interference of mevalonate pathway enzymes. (**A**) BESC isolated from 3 animals, and (**B**) HESC from 3 independent passages, were transfected with scramble siRNA or siRNA targeting *HMGCR*, *FDPS* or *FDFT1*. The mRNA expression of each cognate gene was measured by qPCR, and data presented as mean (SEM) relative to two reference genes. Data were analyzed by Student’s t-test; values differ from scramble, **P < 0.01, ***P < 0.001. BESC isolated from 3 animals (**C**,**E**,**G**) or HESC from 3 independent passages (**D**,**F**,**H**) were incubated for 48 h in serum-free control medium, or media containing scramble siRNA or siRNA targeting *HMGCR, FDPS* or *FDFT1*, or cultured with methyl-β-cyclodextrin (MBCD) as a positive control. Cellular cholesterol (**C**,**D**) was measured and normalized to phospholipid concentrations, and data presented as arbitrary units (AU) with each dot representing an independent animal or cell passage, and a horizontal line indicating the mean. Cells were challenged with control media (■) or media containing PLO () using 100 HU/ml for BESC and 200 HU/ml for HESC, for 2 h. Cell viability was quantified by MTT assay (**E**,**F**), and supernatants were collected to measure LDH (**G**,**H**). Data are presented as mean (SEM), and were analyzed by one-way ANOVA and Dunnett’s multiple comparison post-hoc test; values differ from scramble challenged with PLO,*P < 0.05, **P < 0.01, ***P < 0.001.

We used RNA interference and pharmaceutical inhibitors to explore the role of the mevalonate pathway in cellular resilience to PLO. In the first approach, BESC and HESC were transfected with short interfering RNA (siRNA) sequences targeting key genes in the mevalonate pathway *HMGCR, FDPS* and *FDFT1* (Table 1). The transfection and RNA interference technique in serum-free media did not significantly affect cell viability or cell permeability, as determined by MTT and LDH assays, respectively (Supplementary Fig. S2). However, mRNA expression was reduced by 48%, 90% and 69% with siRNA targeting *HMGCR, FDPS* and *FDFT1*, respectively, in BESC (Fig. 3A), and by 91%, 89% and 91%, respectively, in HESC (Fig. 3B). Cellular concentrations of cholesterol were also measured to quantify the effectiveness of the siRNA. Each of the siRNA significantly reduced the concentrations of cholesterol in BESC and HESC, and the concentrations were similar to cells treated with MBCD to deplete cholesterol (Fig. 3C,D).

**Table 1:** siRNA sequence for target gene knockdown.

Species	Gene	Direction	Sequence (5′ → 3′)
*Bos taurus*	*HMCGR*	Sense	CAGCAUGGAUAUUGAACAAUU
		Antisense	UUGUUCAAUAUCCAUGCUG
*Bos taurus*	*FDPS*	Sense	GCACAGACAUCCAGGACAAUU
		Antisense	UUGUCCUGGAUGUCUGUGCUU
*Bos taurus*	*FDFT1*	Sense	GCGAGAAGGGAGAGAGUUUUU
		Antisense	AAACUCUCUCCCUUCUCGC
*Homo sapiens*	*HMCGR*	Sense	GGAUAAACCGAGAAAGAAAUU
		Antisense	UUUCUUUCUCGGUUUAUCC
*Homo sapiens*	*FDPS*	Sense	GCAGAAGGAGGCUGAGAAAUU
		Antisense	UUUCUCAGCCUCCUUCUGC
*Homo sapiens*	*FDFT1*	Sense	GCAAGGAGGAAGAGAGUUCUU
		Antisense	GAACUCUCUUCCUCCUUGC

The BESC and HESC were next transfected with siRNA for 48 h and then challenged with PLO for 2 h. Cells transfected with control scramble siRNA had a reduction in cell viability when challenged with PLO, as determined by MTT assay (Fig. 3E,F), and increased accumulation of LDH in supernatants (Fig. 3G,H). Using MBCD to deplete cellular cholesterol as a positive control reduced the impact of PLO in BESC and HESC. As might be expected with primary cells, the effect of siRNA in BESC was somewhat variable. Although siRNA targeting *FDPS* did not significantly rescue BESC when challenged with PLO, depleting *HMGCR* and *FDFT1* increased cell resilience to PLO (Fig. 3E). However, depletion of *HMGCR*, *FDPS* and *FDFT1* did not significantly change the BESC leakage of LDH when challenged with PLO (Fig. 3G). In HESC, only siRNA depletion of *FDFT1* significantly increased cell resilience to PLO and reduced leakage of LDH (Fig. 3G,H). Together these data provide evidence that siRNA targeting mevalonate pathway enzymes reduces cellular cholesterol, but only depletion of specific enzymes, such as FDFT1, enhances stromal cell resilience to PLO.

### Inhibitors of mevalonate pathway enzymes enhance cellular resilience to PLO

**Figure 4:**
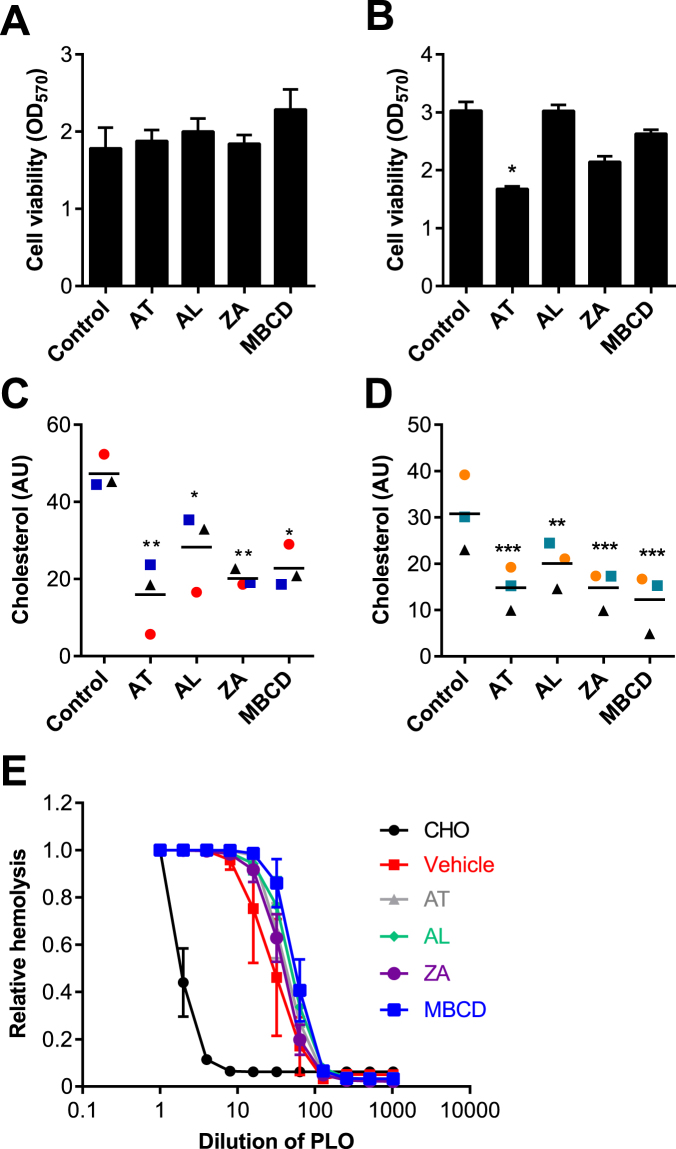
Inhibition of mevalonate pathway enzymes. (**A**,**C**) BESC from 3 animals and (**B**,**D**) HESC from 3 independent passages were incubated in control serum-free medium, or media containing 1 μM atorvastatin (AT), 10 μM alendronate (AL), 10 μM zaragozic acid (ZA) or methyl-β-cyclodextrin (MBCD) for 48 h. (**A**,**B**) Cell viability was assessed by MTT assay. Data are expressed as mean (SEM), and were analyzed by one-way ANOVA and Dunnett’s multiple comparison post hoc test; values differ from control, *P < 0.05. (**C**,**D**) Cellular cholesterol was measured and normalized to phospholipid concentrations, and data presented as arbitrary units (AU) with each dot representing an independent animal or cell passage, and a horizontal line indicating the mean. Data were analyzed by one-way ANOVA and Dunnett’s multiple comparison post hoc test; values differ from control, *P < 0.05, **P < 0.01 and ***P < 0.001. (**E**) The inhibitors or vehicle were incubated with 100 HU/ml PLO for 1 h in DPBS, using 1 mM cholesterol (CHO) as a positive control and DPBS as a negative control (Vehicle), and then incubated with 0.5% (v/v) horse red blood cells for 1 h at 37 °C to assess hemolysis. Data are expressed as mean (SEM) relative hemolysis of three independent experiments.

For our second approach to explore cellular resilience to PLO, we selected atorvastatin to inhibit HMGCR, the bisphosphonate alendronate to inhibit FDPS, and zaragozic acid to inhibit FDFT1. In order to determine if the inhibitors might reduce cell viability *per se*, BESC and HESC were incubated with the manufacturer’s recommended concentrations of 1 μM atorvastatin, 10 μM alendronate and 10 μM zaragozic acid for 48 h in serum free media, prior to evaluating cell viability using the MTT assay. Apart from atorvastatin in HESC, the inhibitors did not significantly reduce BESC or HESC viability (Fig. 4A,B). However, each of the inhibitors reduced cellular cholesterol in BESC and HESC, compared with control (Fig. 4C,D).

One concern with the inhibitors was that observations might be misleading if the inhibitors could bind PLO. To test this, each inhibitor was incubated with PLO for 1 h prior to addition of horse red blood cells and measurement of hemolysis. Cholesterol was used as a positive control to bind PLO and reduce hemolysis. However, the mevalonate pathway inhibitors or MBCD did not significantly alter the hemolytic effect of PLO (Fig. 4E).

**Figure 5:**
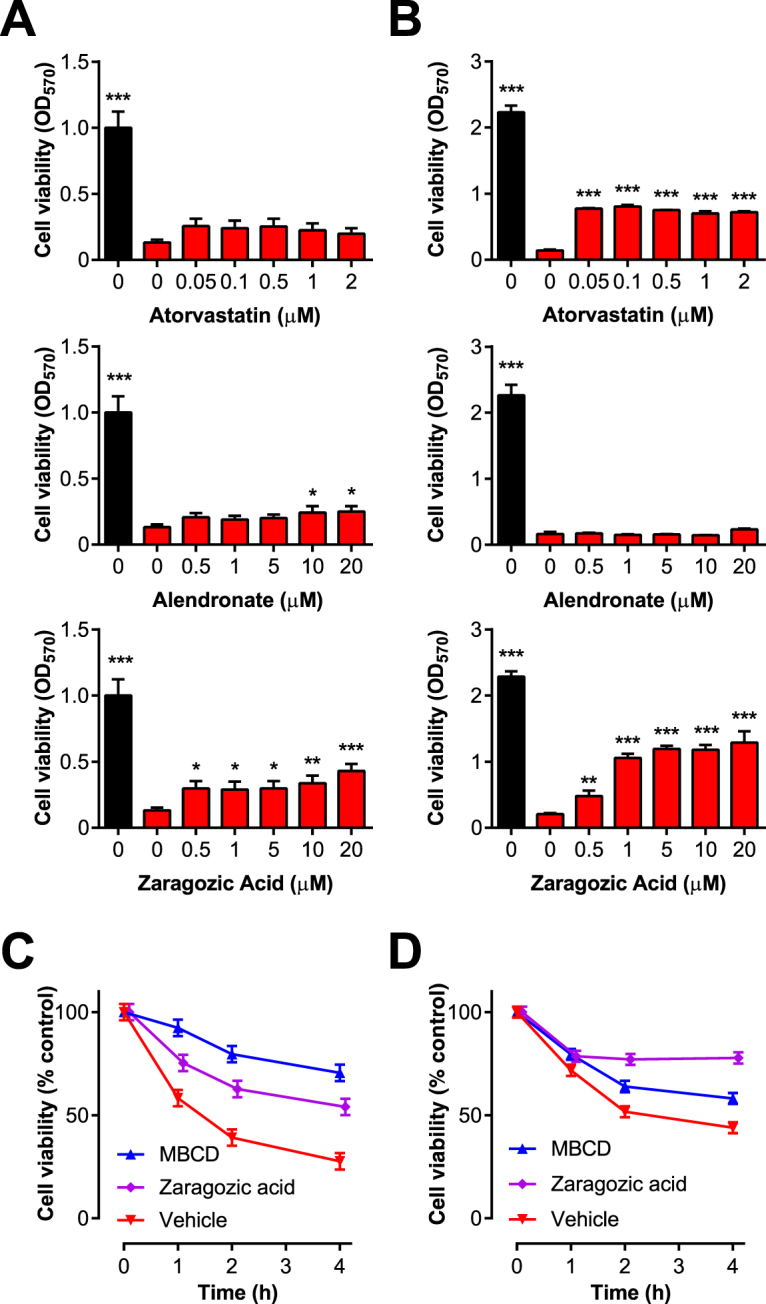
Mevalonate pathway inhibitors modulate the impact of PLO on cell viability. (**A**) BESC isolated from 4 animals, or (**B**) HESC from 3 independent passages were incubated for 48 h with control serum-free medium, or media containing the indicated concentrations of atorvastatin, alendronate or zaragozic acid, and then challenged with control media (■) or media containing PLO () using 100 HU/ml for BESC and 200 HU/ml for HESC, for 2 h. Cell viability was determined by MTT assay. Data are expressed as mean (SEM), and were analyzed by one-way ANOVA with Dunnett’s multiple comparison post hoc test; values differ from PLO treatment with no inhibitor, *P < 0.05, **P < 0.01, ***P < 0.001. In independent experiments, BESC isolated from 4 animals were incubated for (**C**) 24 h or (**D**) 48 h in serum-free media containing vehicle, MBCD or 10 μM zaragozic acid. Cells were then challenged with 100 HU/ml PLO, and cell viability was monitored every hour using the Alamar Blue assay. Data are expressed as mean (SEM) percentage viability of control.

Having established the mevalonate pathway inhibitors could reduce cellular cholesterol, BESC and HESC were incubated for 48 h with a range of concentrations of atorvastatin, alendronate or zaragozic acid, prior to challenge with PLO for 2 h. In BESC, there was no significant effect of atorvastatin, but alendronate and particularly zaragozic acid increased the resilience of cells to PLO (Fig. 5A). In HESC, there was no significant effect of alendronate, but atorvastatin and particularly zaragozic acid increased the resilience of cells to PLO (Fig. 5B). To further examine the effect of zaragozic acid, BESC were treated for 24 h or 48 h with 10 μM zaragozic acid prior to challenge with PLO, and cell viability monitored hourly using the Alamar Blue assay^34^, which depends on the cellular enzymatic reduction of resazurin to resorufin (Fig. 5C,D). Cellular resilience to PLO was increased by 24 h or 48 h treatment with zaragozic acid (ANOVA, P < 0.05, and P < 0.001, respectively).

**Figure 6:**
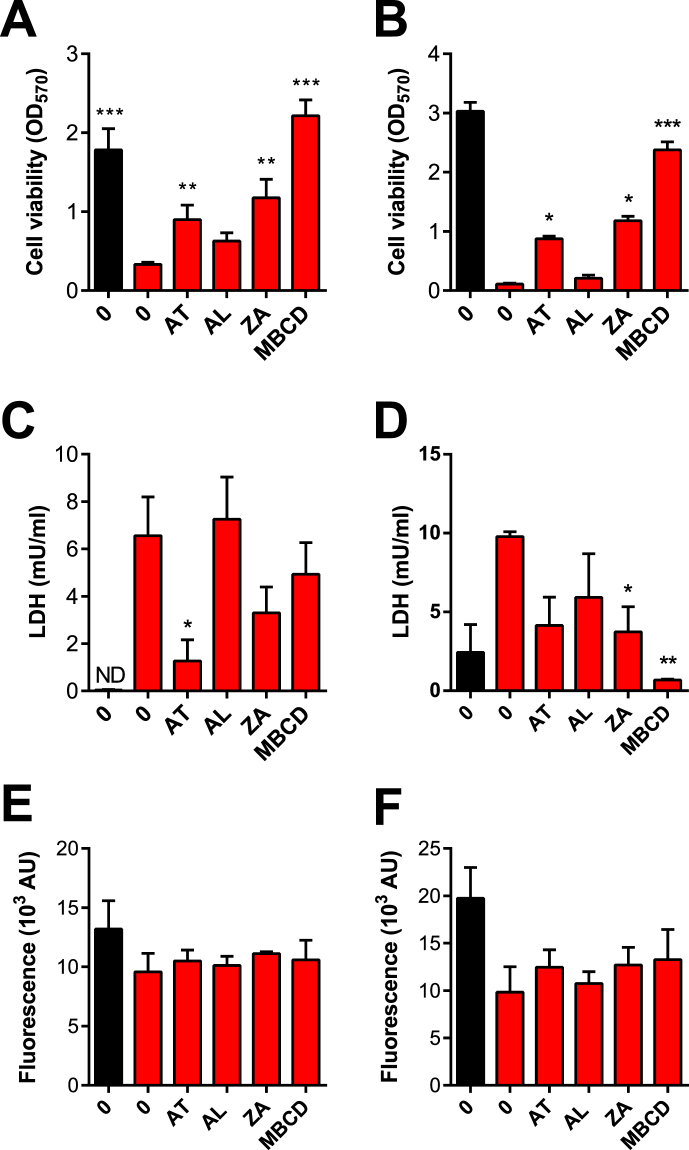
Mevalonate pathway inhibitors and cellular resilience to PLO. (**A**,**C**,**E**) BESC from 3 animals, and (**B**,**D**,**F**) HESC from 3 independent passages were incubated with control serum-free media (0), or media containing 1 μM atorvastatin (AT), 10 μM alendronate (AL), 10 μM zaragozic acid (ZA) or methyl-β-cyclodextrin (MBCD) for 48 h, and then challenged with control media (■) or media containing PLO () using 100 HU/ml for BESC and 200 HU/ml for HESC, for 2 h. Cell viability was evaluated by MTT assay (**A, B**), supernatants were collected for LDH assay (**C**,**D**), and total cell DNA was determined by CyQuant assay (**E**,**F**). Data are expressed as mean (SEM), and were analyzed by one-way ANOVA with Dunnett’s multiple comparison post hoc test; values differ from PLO treatment with no inhibitor, *P** < **0.05, **P < 0.01 and ***P < 0.001; ND, below limits of detection.

To further explore the impact of mevalonate pathway inhibitors, BESC and HESC were incubated with control serum-free media, or media containing 1 μM atorvastatin, 10 μM alendronate, 10 μM zaragozic acid, or 1 mM methyl-β-cyclodextrin (MBCD) for 48 h, and then challenged with control medium or PLO for 2 h. The inhibitors *per se* did not significantly affect cell viability or the release of LDH in control medium (all values within 10% of control). However, atorvastatin and zaragozic acid reduced the impact of PLO on cell viability, as determined by MTT assay, in BESC (Fig. 6A) and HESC (Fig. 6B). There was also a significant reduction in the leakage of LDH for atorvastatin in BESC, and zaragozic acid in HESC (Fig. 6C,D). To determine whether the protection against PLO by mevalonate pathway inhibitors affected the number of cells, total cellular DNA was measured using the CyQuant assay. Total cellular DNA remained unchanged after challenge with control media or PLO (Fig. 6E,F), indicating that changes in viability were independent of the number of cells. Taken together, these data support a concept that the resilience of stromal cells to PLO is increased by inhibiting specific mevalonate pathway enzymes, particularly FDFT1.

## Discussion

We found that inhibiting enzymes in the mevalonate pathway reduced cholesterol abundance in both primary bovine endometrial stromal cells (BESC) and telomerase-immortalized human endometrial stromal cells (HESC). However, surprisingly, inhibiting each mevalonate pathway enzyme did not equally affect cellular resilience to PLO. Inhibiting FDFT1 most consistently increased stromal cell resilience to PLO. We suggest that manipulating the mevalonate pathway may help tissue cells tolerate bacterial infections. We also speculate that topical application of FDFT1 inhibitors could be used therapeutically in the uterus.

Pyolysin is considered to be the only cytolysin secreted by *T. pyogenes*, as determined using an anti-PLO antibody or deletion of the *plo* gene^10,16,35,36^. To examine mechanisms of cellular resilience, the present study used stromal cells because it is important to use tissue cells that could influence the ability of tissues to tolerate pathogens^2,4^. As expected, BESC were sensitive to recombinant PLO protein^16,20^. However, PLO also reduced cellular viability and increased the leakage of LDH into supernatants of HESC. The sensitivity of HESC to PLO was unexpected as *T. pyogenes* only rarely causes disease in humans^37^. However, the potency of PLO across species and tissues may help explain the wide diversity of species and diseases that are associated with *T. pyogenes* infection. Although a specific cell surface receptor cannot be ruled out, the ability of exogenous cholesterol to inhibit hemolysis in the present study and the sensitivity of HESC to PLO, provides evidence that cholesterol is likely the main target of PLO.

Plasma membranes of cultured fibroblast cells from human foreskin contain half their cellular phospholipid and 90% of their cholesterol and sphingomyelin^7^. In the plasma membrane, cholesterol is distributed amongst an essential pool, a sphingomyelin sequestered pool, and a labile pool of cholesterol that binds to cholesterol-dependent cytolysins^17^. We assume that this labile pool of cholesterol is most likely depleted when cells are treated with MBCD^18^. In the present study, MBCD consistently reduced cellular cholesterol abundance in BESC and HESC, and this was associated with increased resilience to PLO. Although MBCD has not been used with HESC previously, these observations are consistent with findings for BESC and other cells, and with a range of cholesterol-dependent cytolysins^6,16,20,33^. Cellular cholesterol abundance depends on cholesterol uptake from LDL, cholesterol efflux, and cholesterol synthesis via the mevalonate pathway^8,9^. Cholesterol homeostasis is highly regulated in cells and HMGCR is usually considered to be the rate-limiting enzyme in cholesterol synthesis, although this may not apply to all cells^21,22^. The importance of the mevalonate pathway for cellular cholesterol was verified in the present study, using siRNA or inhibitors to target HMGCR, FDPS, and FDFT1 in endometrial stromal cells, which consistently reduced cellular cholesterol by about half; similar to the effect of MBCD.

The surprising finding in the present study was that despite siRNA and inhibitors reducing cellular cholesterol, the effect on cellular resilience varied. Depleting *FDFT1* mRNA expression or inhibiting FDFT1 activity with zaragozic acid most effectively increased cellular resilience to PLO in BESC and HESC. Thus, factors other than the abundance of cholesterol influenced the ability of cells to tolerate PLO. One possible explanation is that off-target effects of the inhibitors could affect cell resilience, although this seems less likely for siRNA. Another possibility is that while inhibition of each enzyme reduces cholesterol synthesis, the abundance of substrates and products will likely vary for each enzyme. For example, inhibiting FDFT1 may lead to increased farnesyl pyrophosphate, whereas inhibiting HMGCR would likely reduce farnesyl pyrophosphate concentrations. In addition, there may be wider impacts on the pathways that regulate cholesterol homeostasis, such as sterol regulatory element binding proteins^21^. Indeed, activation of lipogenic genes by sterol regulatory element binding protein 1 promoted cell survival after toxin attack^38^.

A limitation of the present study was that BESC show some variation in their susceptibility to PLO, as determined by MTT assay or LDH leakage, and the effects of modulating the mevalonate pathway. Isolation and culture of primary bovine endometrial stromal cells was performed as described previously^39,40^. Some of the variance may reflect differences amongst the animals from which the cells were isolated, and biological variation is a consistent feature of primary endometrial stromal cells function determined by MTT, LDH and cholesterol assays^16,20,39^. In addition, as BESC and HESC continue to grow during experiments, the OD_570_ values from the MTT assay also increase when there is a longer duration of the experiment. To provide additional evidence for our claims, future work might use more animals to isolate cells, a wider range of measurements of cell resilience, and use *in vivo* experiments.

The ability to increase tissue cell resilience so that tissues can tolerate pathogens is important for health^2,4^. Several cellular pathways increase the resilience of cells to cholesterol-dependent cytolysins, including mitogen-activated protein kinases, the unfolded protein response, autophagy, calcium ion flux, and caspase-1^38,41–44^. Unfortunately, manipulating these pathways to protect against cholesterol-dependent cytolysins *in vivo* may be challenging because inhibitors of these pathways are often toxic. However, statins are widely used *in vivo*, and simvastatin enhanced cellular resistance against the cholesterol-dependent cytolysin pneumolysin in human endothelial cells^45^. Similarly, simvastatin or siRNA targeting HMGCR protected human airway epithelial cells against pneumolysin^29^. Interestingly, and in accord with our findings, the protective mechanism of simvastatin was not solely mediated by reducing cellular cholesterol^29^. Furthermore, Statt *et al*. (2015) also found that an inhibitor of FDFT1 or siRNA depleting *FDFT1* mRNA reduced the impact of pneumolysin on cells^29^. In the present study, zaragozic acid increased the resilience of both BESC and HESC to PLO, and was not toxic to cells at the concentrations we used.

Interestingly, zaragozic acid also reduces BESC inflammatory responses to endotoxin from Gram-negative bacteria^46^; and, reduces serum cholesterol concentrations *in vivo* ^25^. Thus, zaragozic acid might be worthy of further investigation to increase tissue tolerance to bacteria. One therapeutic advantage with uterine infections is that treatments are often topical applications, via intrauterine infusion, which provides high concentration of the pharmaceutical molecule with less risk of toxicity^11^. We suggest that intrauterine infusion of inhibitors targeting mevalonate pathway enzymes could be used to limit the severity of uterine disease caused by *T. pyogenes* in postpartum animals. An alternative approach would be to infuse MBCD or other cyclodextrins that deplete cholesterol. However, cyclodextrins can also act as cholesterol donors, with the danger of toxicity^19^.

In conclusion, the present study provides evidence that manipulating the mevalonate pathway increases the resilience of cells to PLO, to help tissues tolerate bacterial infections. We speculate that inhibitors of the mevalonate pathway could be used to increase cellular resilience to cholesterol-dependent cytolysins, and limit the severity of disease.

## Methods

### Pyolysin

The PLO plasmid (pGS59) was a generous gift from Prof BH Jost (University of Arizona, USA), and the DS-PLO mutant plasmid (R219C/G85C) was a kind gift from Prof M Palmer (University of Waterloo, Canada). Proteins were generated as described previously^16,27,28^. The abundance of proteins was measured by DC assay, and their purity evaluated using SDS-PAGE and Coomassie blue staining, as described previously^16^.

A hemolysis assay was used to determine the activity of PLO, as described previously^16^. Optical density (OD_450_) was measured using a microplate reader (POLARstar Omega; BMG Labtech, Offenburg, Germany). Hemolytic units were mathematically determined by 4-parameter modelling using the Solver function in Microsoft Excel. Endotoxin concentrations in stock solutions of PLO were all <1 EU/ml, as determined by LAL assay (LAL endotoxin quantitation kit; Thermo Scientific, Hertfordshire, UK). To examine the potential binding to PLO, 1 μM atorvastatin, 10 μM alendronate, 10 μM zaragozic acid, 1 mM methyl-β-cyclodextrin, and 1 mM cholesterol (all Sigma) were incubated with 100 HU/ml PLO for 1 h, prior to conducting the hemolysis assay.

### Ethical statement

No live animal experiments were conducted. Uteri were collected from cattle after they were slaughtered and processed as part of the normal work of an abattoir.

### Cell culture

Isolation and culture of primary bovine endometrial stromal cells was performed as described previously^39,40^. Briefly, uteri with no gross evidence of genital disease or microbial infections were collected from cattle after they were slaughtered and processed as part of the normal work of an abattoir. No live animal experiments were used. Stromal cells were isolated by enzymatic digestion of the endometrium, sieving the cell suspension through a 40-μm mesh, and differential adhesion to cell culture plates^39,40^. Stromal cells were maintained in BESC complete culture medium comprising RPMI-1640 (Gibco, Gaithersburg, MD, USA) with 10% fetal bovine serum, and 50 IU/ml penicillin, 50 µg/ml streptomycin and 2.5 µg/ml amphotericin B (all Sigma). Cells were incubated at 37°C in humidified air with 5% CO_2_.

The HESC cells were generously gifted by Dr G Krikun (Yale University School of Medicine, USA; ATCC cell line CRL-4003) and were cultured as described previously^26^. Briefly, cells were grown in 75 cm^2^ tissue culture flasks containing HESC complete culture medium, comprising a 1:1 mixture of Dulbecco’s modified Eagle’s medium and Ham’s F-12 medium (Gibco), supplemented with 12% fetal bovine serum, 1% insulin-transferrin-selenium-ethanolamine (ITS-X; Gibco), 50 IU/ml penicillin, 50 µg/ml streptomycin, and 2.5 µg/ml amphotericin B. Cells were incubated at 37°C in humidified air with 5% CO_2_, and used for up to 30 passages. The identity of HESC was verified at the end of the project by short tandem repeat profiling (LGC Standards, Teddington, UK; FTA barcode STRA1969; exact match to ATCC human cell line CRL-4003).

### Cell experiments

To examine cellular resilience, BESC and HESC were seeded at a density of 50,000 cells per well in 24-well tissue culture plates (TPP, Trasadingen, Switzerland), and were cultured for 24 h in their respective complete culture media. Cells were then incubated in serum free media with atorvastatin, alendronate or zaragozic acid, using the concentrations and durations indicated in *Results*, prior to challenge with control medium or medium containing PLO. Methyl-β-cyclodextrin was used to deplete cellular cholesterol, using 0.5 mM for BESC and 1 mM for HESC. Alternatively, BESC and HESC were transfected with scramble siRNA (ON-TARGETplus Non-targeting siRNA #1; Dharmacon; gelifesciences.com) or siRNA targeting *HMCGR*, *FDPS* or *FDFT1*, which were designed using Dharmacon’s siDESIGN Center (Table 1). Briefly, duplex complexes were formed by adding 100 pM of siRNA to 500 μl/well Opti-MEM I medium (Invitrogen, Waltham, MA) in 6-well plates (TPP), and then adding 7.5 μl Lipofectamine RNAiMAX Reagent (Invitrogen). Following 20 min incubation at room temperature, 250,000 exponentially growing cells were then seeded in 2.5 ml/well of their respective complete media for 48 h, prior to challenge with control medium or medium containing PLO.

### Cell viability assays

Cell viability was assessed by the mitochondria-dependent reduction of 3-(4,5-dimethylthiazol-2-yl)-2,5-diphenyltetrazolium bromide (MTT, Sigma) to formazan, as described previously^30^. Briefly, once supernatants were collected, the remaining cells were incubated for 2 h in 250 μl/well serum-free culture medium containing 1 mg/ml MTT; the medium was then removed and the cells lysed with 250 μl/well dimethyl sulfoxide (Sigma); and, optical density (OD_570_) measured using a POLARstar Omega microplate reader.

The kinetics of cell survival was examined by enzymatic reduction of resazurin to resorufin using the Alamar Blue cell viability assay (Pierce, Rockford, IL, USA)^34^. Fluorescence was measured at 545/590 nm (excitation/emission) using a POLARstar Omega microplate reader, with data expressed as the percentage of control cell survival.

Total cellular DNA concentrations were measured using the CyQuant Cell Proliferation Assay (Thermo Fisher Scientific, Loughborough, UK). Fluorescence was measured at 480/530 nm (excitation/emission), with data expressed as arbitrary fluorescence units.

Leakage of cellular LDH was measured in cell culture supernatants using the Lactate Dehydrogenase Activity Colorimetric Assay Kit (Biovision, California, USA), according to the manufacturer’s instructions.

### Cholesterol measurement

The BESC and HESC were grown at a density of 100,000 cells/well in 12-well tissue culture plates and treated with siRNA or inhibitors, as described above. After the treatment period, the cells were collected in 200 µl/well 1X cholesterol assay buffer and stored in Eppendorf tubes at −20°C. When needed, the samples were defrosted at room temperature and sonicated for 10 min in a sonicating water bath. Cellular cholesterol content was measured using the Amplex® Red Cholesterol Assay Kit (Invivogen), according to the manufacturer’s guidelines. Total cellular phospholipid was measured in the samples prepared for the cholesterol assay using a phospholipid assay kit (Sigma Aldrich, MAK122), according to the manufacturer’s guidelines. The cholesterol concentrations were then normalized to the phospholipid concentrations, and the data are expressed as arbitrary units.

### Quantitative PCR (qPCR)

Cellular RNA was extracted using the RNeasy Mini Kit according to the manufacturer’s instructions (Qiagen, Crawley, UK). The RNA was quantified using a Nanodrop ND1000 spectrophotometer (Labtech, Ringmer, UK), and 1 μg of total RNA added to a genomic DNA elimination reaction, followed by conversion to cDNA (Quantitect Reverse Transcription Kit, Qiagen), according to the manufacturer’s instructions. Quantitative PCR was performed using intron-spanning primers (Table 2) and the IQ5 system (Bio-Rad, Hemel Hempstead, UK). The starting quantity of mRNA was determined using standard curves generated from serial dilutions of pooled reference RNA with Quantifast SYBR green (Qiagen). The target and reference genes were analyzed in triplicate, and mRNA expression normalized to the *ACTB* and *RPL19* reference genes (Table 2) using the IQ5 system (Bio-Rad), with inter-run correlation and run-dependent differences corrected using qBase software on the IQ5 system (Bio-Rad)^47^. The reference genes did not differ in expression with treatment, and were amplified at the same efficiency as the target genes.

**Table 2:** Sequence of target and house-keeping primers.

Species	Gene	Direction	Sequence (5′ → 3′)
*Bos taurus*	*ACTB*	Forward	CAGAAGGACTCGTACGTGGG
		Reverse	TTGGCCTTGGGGTTCAGGG
*Bos taurus*	*RLP19*	Forward	TGTTTTTCCGGCATCGAGCCCG
		Reverse	ATGCCAACTCCCGCCAGCAGAT
*Bos taurus*	*HMCGR*	Forward	TGAGATCCGGAGGATCCGAG
		Reverse	CAGATGGTCAGCGTCACTGT
*Bos taurus*	*FDPS*	Forward	ATGACGGGTAAGATCGGCAC
		Reverse	TTCTGCCCATAGTTCTCCTGC
*Bos taurus*	*FDFT1*	Forward	GGCACCCTGAGGAGTTCTAC
		Reverse	GCATACTGCATGGCGCATTT
*Homo sapiens*	*ACTB*	Forward	ATGATGATATCGCCGCGCTC
		Reverse	CCACCATCACGCCCTGG
*Homo sapiens*	*RLP19*	Forward	GCGAGCTCTTTCCTTTCGCT
		Reverse	TGCTGACGGGAGTTGGCATT
*Homo sapiens*	*HMCGR*	Forward	GTTAACTGGAGCCAGGCTGA
		Reverse	CCTTGGATCCTCCAGATCTCAC
*Homo sapiens*	*FDPS*	Forward	GAGACCGGGCCTTACTTCTG
		Reverse	GGACAGGGGCATCCTTCAAA
*Homo sapiens*	*FDFT1*	Forward	GACTCGACAGACTCTAAGGCTC
		Reverse	TGGTCAATAAGTCGCCCACG

### Statistical analysis

Data are presented as arithmetic mean and error bars represent SEM. The statistical unit was designated as the animal for BESC, and each independent cell passage for HESC. Statistical analysis was performed using Graphpad Prism 5.0.1 and SPSS 20.0, with P < 0.05 considered statistically significant. Comparisons between two treatments were tested using Student’s t-test, and amongst several treatments using ANOVA, followed by Dunnett’s post hoc multiple comparison test.

### Data availability

All data generated or analyzed during this study are included in this published article (and its Supplementary Information files).

## Electronic supplementary material

Supplementary Information
