# Supplementary material for: Inhibiting mevalonate pathway enzymes increases stromal cell resilience to a cholesterol-dependent cytolysin
Source: Sci Rep. 2017 Dec 6;7:17050. doi: 10.1038/s41598-017-17138-y (PMC5719056; doi:10.1038/s41598-017-17138-y)
Supplement: Supplementary file 1 — Supplementary Information [file 41598_2017_17138_MOESM1_ESM.pdf]

Supplementary Information

Inhibiting mevalonate pathway enzymes increases stromal cell resilience to a cholesterol-dependent cytolysin

Sholeem Griffin[1], Giulio Preta[1,2], Iain Martin Sheldon[1]*

[1]Swansea University Medical School, Swansea University, Swansea, SA2 8PP, United Kingdom

[2]Institute of Biochemistry, Life Science Center, Vilnius University, Vilnius, Lithuania.

*Address correspondence to: Prof Iain Martin Sheldon, Institute of Life Science, College of Medicine, Swansea University, Singleton Park, Swansea, SA2 8PP, United Kingdom. E-mail: i.m.sheldon@swansea.ac.uk

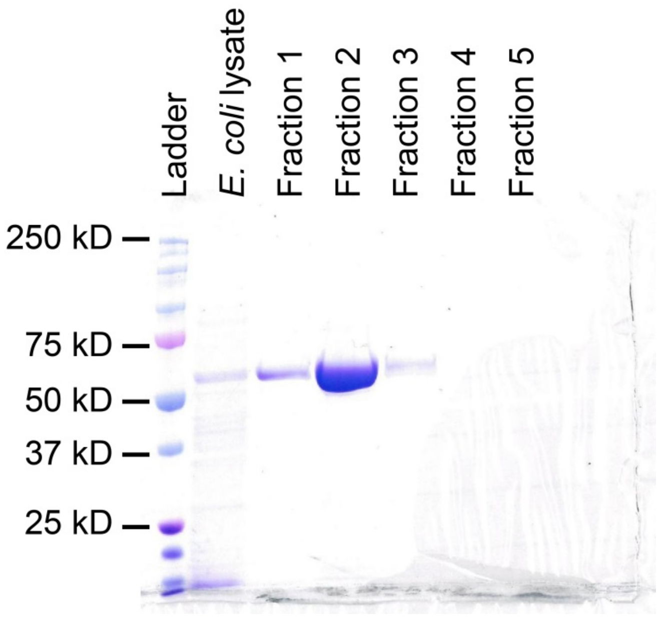

Supplementary Figure S1. The purity of PLO protein. The purity of recombinant PLO protein produced in *Escherichia coli* was evaluated using SDS-PAGE and Coomassie blue staining. Soluble fractions were diluted to 20 μg/μl in a 1:1 ratio with Laemelli sample buffer and heated to 95˚C for 10 min before loading onto 12% Mini-PROTEAN® TGX™ precast gel. A Dual colour standard ladder was used as marker, and a sample volume of 20 μl was loaded in each well and run at 120V until the dye front reached the bottom of the gel. SDS-PAGE gels were stained overnight in Coomassie stain (50% (v/v) dH2O, 40 (v/v) methanol, 10% (v/v) acetic acid and 0.5% (w/v) Coomassie blue R250), and destained with destain solution (50% (v/v) dH_2O, 40 (v/v) methanol and 10% (v/v) acetic acid) until clear. The second fraction was used in the present studies.

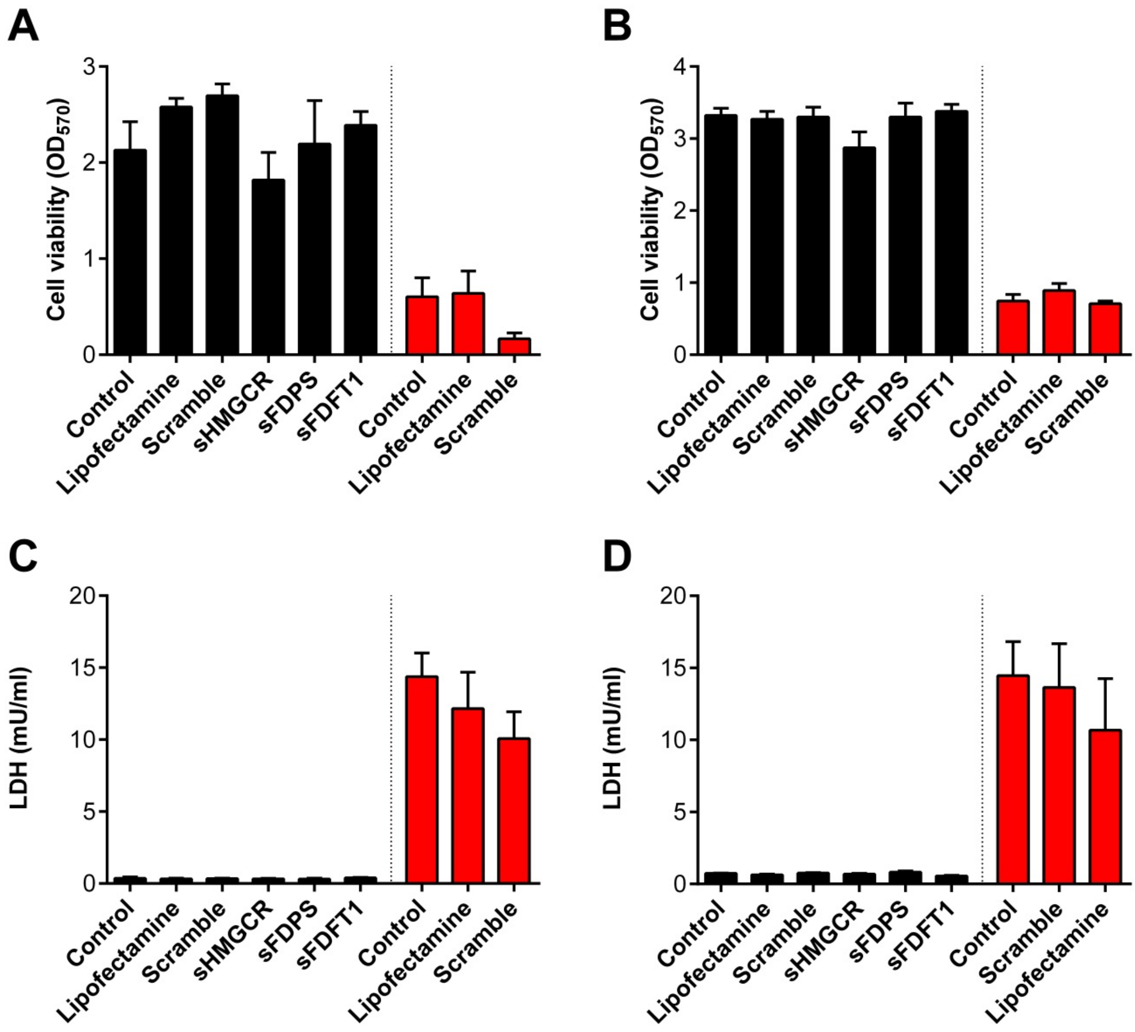

Supplementary Figure S2. RNA interference of BESC and HESC. The effect of siRNA transfection on cell viability was determined by incubating BESC from 4 animals (A, C) or HESC from 4 independent passages (B, D) for 48 h in control medium, medium containing lipofectamine, or transfecting cells with scramble siRNA or siRNA targeted against *HMGCR*, *FDPS* or *FDFT1*. Cells were challenged with control media (black bars) or media containing PLO (red bars) using 100 HU for BESC and 200 HU for HESC, for 2 h. Cell viability was determined by MTT assay (A, B), and cell permeability by measuring LDH in cell supernatants (C, D). Data are expressed as mean (SEM).
